# Preparation and performance of a colorimetric biosensor using acetylcholinesterase and indoxylacetate for assay of nerve agents and drugs

**DOI:** 10.2478/intox-2014-0031

**Published:** 2015-03-04

**Authors:** Miroslav Pohanka, Vitezslav Vlcek

**Affiliations:** 1 Faculty of Military Health Sciences, University of Defence, Trebesska 1575, 500 01 Hradec Kralove, Czech Republic; 2 Faculty of Agronomy, Mendel University in Brno, Zemedelska 1, 613 00 Brno, Czech Republic

**Keywords:** acetylcholinesterase, pesticide, inhibitor, indoxylacetate, Ellman′s method, carbofuran, sarin, rivastigmine

## Abstract

Different toxic compounds can target the cholinergic nervous system. Acetylcholinesterase (AChE; EC 3.1.1.7) is one of the most crucial components of the cholinergic nervous system and thus many of the toxins interact with this enzyme. As to inhibitors, nerve agents used as chemical warfare, some insecticides, and drugs influencing the cholinergic system are common examples of AChE inhibitors. Once inhibited by a neurotoxic compound, a serious cholinergic crisis can occur. On the other hand, sensitivity of AChE to the inhibition can be used for analytical purposes. In this study, a simple disposable biosensor with AChE as a recognition element was devised. AChE was immobilized onto a cellulose matrix and indoxylacetate was used as a chromogenic substrate. The enzyme reaction was assessed by the naked eye using arbitrary units and pyridostigmine, tacrine, paraoxon, carbofuran, soman and VX were assayed as selected inhibitors. A good stability of the biosensors was found, with no aging over a quarter of a year and minimal sensitivity to the interference of organic solvents. The limit of detection ranged from 10 to 100 nmol/L for the compounds tested with a sample volume of 40 µL.

## Introduction

Different toxic compounds can target the cholinergic nervous system. The enzyme acetylcholinesterase (AChE; EC 3.1.1.7) is one of the most crucial components of the cholinergic nervous system. It can be inhibited by many neurotoxic compounds. As to AChE inhibitors, nerve agents used as chemical warfare, some insecticides, and drugs influencing the cholinergic system are common examples. AChE inhibition results in accumulation of acetylcholine in neurosynaptic clefts or neuromuscular junctions (Pohanka, 2011).

The fact that AChE is inhibited by the compounds mentioned was noticed by analytical chemists. Experimental protocols for diagnostic purposes and *in vitro* assays were introduced. The probably best known protocol uses 5,5′-dithio-bis-2-nitrobenzoic acid (DTNB) as a chromogen and acetylthiocholine as a substrate. It was introduced by Ellman and co-workers for diagnostic purposes (Ellman *et al.*, 1961). Many adaptations followed, including construction of biosensors for assaying inhibitors (Miao *et al.*, 2010).

**Figure 1:**
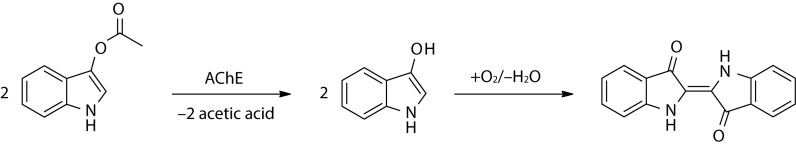
Principle of indoxylacetate splitting catalyzed by AChE and formation of indigo blue.

Assays based on Ellman′s method have some drawbacks, such as instability of substrates and interference of some substances including hemoglobin (Haigh *et al.*, 2008; Pohanka, 2011). Indoxylacetate is another chromogenic substrate suitable for assessment of cholinesterase activity (Pohanka & Drtinova, 2013). The principle of the reaction consists of splitting the acetate moiety from indoxylacetate, followed by spontaneous creation of blue indigo (Figure 1).

In the previous experiment, a colorimetric dipstick based on AChE immobilized on a cellulose matrix was constructed for the assay of neostigmine and paraoxon, using evaluation by the naked eye (Pohanka, 2012). Unfortunately, the reported experiment did not cover assays of highly toxic nerve agents and currently used drugs and pesticides, and thus demonstration of the device function fails to be sufficient. The experiment performed in this study aimed at optimization of the performance of colorimetric biosensors with immobilized AChE. Verification of biosensor function and calibration for the most relevant nerve agents, pesticides and representative cholinergic drugs was performed in the experiment.

## Materials and methods

### Immobilization procedure

AChE from the electric eel (Sigma-Aldrich, Saint Louis, Missouri, USA; specific activity 16.7 µkat/mg protein) was used throughout the experiment. It was dissolved in phosphate buffered saline and the activity was adjusted at 83.4 nkat (5 U) in 20 µL of stock solution. Enzyme activity was measured in the solution containing 1 mmol/L acetylthiocholine chloride and DTNB 0.1 mg/mL, both solved in phosphate buffered saline.

Cellulose medium filter papers (Whatman, Kent, UK) were cut into bands 5 mm wide and 50 mm long. Per one edge of the band, 20µL of the solution containing 5 U of AChE in PBS and 2% (w/w) gelatin was injected. The bands were dried in a wet incubator with temperature adjusted to 37 °C. After drying, the other edge of the band was imbued by 20 µL of 100 mmol/L indoxylacetate in ethanol and let dry at 24 °C.

### Assay procedure

**Figure 2:**
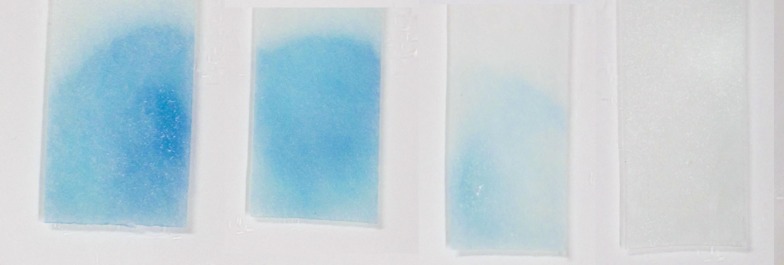
Coloring of biosensors. Equivalent arbitrary units for individual biosensors (from left): +++, ++, +, –.

The compounds assayed were dissolved in 5% 2-propanol (v/v) in a scale of 10^–3^, 10^–4^, 10^–5^, 10^–6^, 10^–7^, 10^–8^ and 10^–9^ mol/L. As a blank, 5% 2-propanol was used. Stability of the dipsticks was examined using ethanol, 2-propanol, dimethyl sulfoxide (DMSO), and the detergent Liqui-nox (Sigma-Aldrich) in a twofold dilution scale: 0.16, 0.32, 0.64, 1.3, 2.5, 5.0, 10, and 20% (v/v) in deionized water. The sample tested was injected in an amount of 40 µL per edge with AChE and incubated for 15 minutes. Then the used dipstick was folded in the middle and the two opposite parts were pressed together to allow distribution of indoxylacetate to the edge with immobilized AChE. Coloration was assessed 30 minutes after the edges were pressed together and scaled using arbitrary units introduced earlier (Pohanka, 2012): – no coloring, + low coloring (light blue), ++ middle coloring (approx. azure blue), +++ intensive coloring (dark blue). Examples of coloring are depicted in Figure 2. All measurements were done in tetraplicate.

### Standards used and manipulation with nerve agents

Pyridostigmine, rivastigmine, tacrine, paraoxon and carbofuran were used as standard drugs or insecticides. They were purchased from Sigma-Aldrich in analytical purity. Soman and VX were obtained from the former Military Technical Institute (Brno, Czech Republic) in at least 95% purity. Manipulation with the nerve agents used was performed in a laboratory under permission of the State Office for Nuclear Safety, a representative of the Czech government for chemical safety. The institution is also responsible to the Organization for the Prohibition of Chemical Weapons (OPCW).

All manipulations with nerve agents were carried out in a specialized digester. The nerve agents were stored in glass tubes placed into a teflon box filled with active carbon. Standard protection suits were used throughout the manipulation. All contaminated tools and disposable laboratory equipment used were decontaminated in 1 mol/L sodium hydroxide with 10% ethanol overnight (soman) or in at least 30% calcium hypochlorite (VX).

## Results and discussion

### Effect of solvents

Freshly prepared biosensors were examined to assess the effect of organic solvents (Table 1). From the solvents tested, 2-propanol and Liqui-nox had no effect even in the highest concentrations used. Ethanol and DMSO inhibited AChE immobilized in the biosensor in the concentration of 20%. Lower concentrations of ethanol and DMSO had no significant impact on the biosensors. The same results were obtained when the experiment was repeated. The examined scale of organic solvents was chosen with regard to pertinent manipulation with assayed unknown samples.

**Table 1:** Effect of organic solvents on prepared biosensors.

Concentration (% v/v)	0.16–10	20
ethanol	+++	++
2-propanol	+++	+++
DMSO	+++	+
Liqui-nox	+++	+++

Arbitrary units: - no coloring, + low coloring, ++ middle coloring, +++ intensive coloring.

The fact that AChE is inhibited by organic solvents is well known. It was reported for membrane-bound AChE (Korpela & Tahti, 1988) and the synergetic effect of organic solvents and pesticides (Turdean & Turdean, 2008). Our finding that the immobilized AChE is well stable once immobilized into a gelatin membrane is contradictory to our previous results (Pohanka, 2013). The immobilization of AChE into gelatin is probably better for stabilizing AChE than the previously used capturing into precipitate by albumin and glutaraldehyde. The findings are in compliance with other experiments, Mionetto *et al.* (1994) reached the same conclusion as we did in this experiment. They recognized significant stabilization of AChE due to immobilization and thus they improving the assay.

### Stability of biosensors

Two experiments were done in order to judge biosensor stability. First, the biosensors were immersed by their edge with AChE into a beaker with deionized water and biosensors were replaced in regular intervals. The stability is presented in Table 2. Biosensors immersed up to 25 minutes had no significant decrease in their coloration when used in the standard manner. Thirty minutes after immersing, a low decrease in intensity of coloration was found after biosensor folding. The results were the same for all repetitions. The experimental data point to a good stability of the prepared biosensors. Owing to the discussion devoted to the stability of biosensors in organic solvents, we infer that the immobilization procedure for biosensor construction is not only able to stabilize AChE, the creating membrane is moreover firm enough to protect the enzyme from washing out when immersed into water. The good stability confirms the suitability of the biosensor for practical performance and assay of unknown samples by immersing the biosensor into them. AChE washing out was reported for some immobilization procedures and it is a limiting step for practical application (Marinov *et al.*, 2009; 2010). We infer that immobilization into gelatin membrane is suitable for biosensor construction regarding the simplicity, price of reagents, and excellent stability of the biorecognition element.

**Table 2:** Stability of biosensors when immersed into water.

Time (minutes)	1–25	30
Arbitrary units	+++	++

Arbitrary units: - no coloring, + low coloring, ++ middle coloring, +++ intensive coloring.

Long-term stability of the prepared biosensors was examined over a four-month interval. For the whole time, biosensors were kept in a dark chamber and standard ambient temperature and pressure (SATP) conditions. Biosensors were used each week for assay of 5% 2-propanol. We recognized no measurable decrease of coloration and the color was scaled by the upper arbitrary unit. The data confirmed the expected excellent long-term stability of the constructed biosensor.

### Calibration

Pyridostigmine, rivastigmine, tacrine, paraoxon, carbofuran, soman and VX were assayed as a solution in 5% 2-propanol. Example of biosensors used for calibration is depicted in Figure 2. Complete calibration data are given in Table 3. The limit of detection was estimated as a shift of one arbitrary unit against the blank. The lowest limit of detection was achieved for tacrine: 10 nmol/l. The last compounds were assayed with a limit of detection of 100 nmol/l. It has to be emphasized that the coloration was assessed by the naked eye. The achieved limits of detection are approximately ten to one hundred times higher than the limits reported in the literature for AChE based biosensors. *E.g.* Du *et al.*, (2010) reached a limit of detection of 2 nmol/l for dimethoate when AChE based electrochemical biosensors with multiwall carbon nanotubes modified glassy carbon electrode was used. A similar limit of detection was reported by Di Tuoro *et al.* (2012): 0.86 ppb for paraoxon (approximately 3.13 nmol/l). The biosensor reported here is a disposable analytical tool for a simple assay of neurotoxic compounds with no specific need to treat the assayed samples. Performance of AChE dipsticks is not a novel idea. *E.g.* Tusarova *et al.* (1999) successfully adopted Ellman′s method for construction of their own dipsticks. The novelty described lies in the immobilization procedure and selection of indoxylacetate as substrate and chromogen.

**Table 3:** Calibration of biosensors.

Concentration (mol/L)	10^–3^	10^–4^	10^–5^	10^–6^	10^–7^	10^–8^	10^–9^	blank
Pyridostigmine	–	–	–	+	++	+++	+++	+++
Rivastigmine	–	–	–	+	++	+++	+++	+++
Tacrine	–	–	–	+	+	++	+++	+++
Paraoxon	–	+	++	++	++	+++	+++	+++
Carbofuran	–	–	+	++	++	+++	+++	+++
Soman	–	–	–	+	++	+++	+++	+++
VX	–	–	+	++	++	+++	+++	+++

Arbitrary units: - no coloring, + low coloring, ++ middle coloring, +++ intensive coloring.

## Conclusions

A disposable colorimetric biosensor was developed. It is suitable for simple assay of neurotoxic compounds including highly toxic nerve agents. The biosensor is well stable when stored or exposed to unfavorable chemical environment. Owing to the practical significance, further development is expected on the issue of AChE and indoxylacetate based dipsticks.
